# Development of Spatial Memory: A Behavioral Study

**DOI:** 10.3390/neurosci5040050

**Published:** 2024-12-19

**Authors:** Konstantinos Kostakos, Alexandra Pliakopanou, Vasileios Meimaridis, Ourania-Natalia (Oriana) Galanou, Aikaterini Argyro Anagnostou, Dimitra Sertidou, Panagiotis Katis, Periklis Anastasiou, Konstantinos Katsoulidis, Yannis Lykogiorgos, Dimitrios Mytilinaios, Andreas P. Katsenos, Yannis V. Simos, Stefanos Bellos, Spyridon Konitsiotis, Dimitrios Peschos, Konstantinos I. Tsamis

**Affiliations:** 1 Department of Physiology, Faculty of Medicine, School of Health Sciences, University of Ioannina, 45110 Ioannina, Greece; 2 Department of Electrical and Computer Engineering, University of Western Macedonia, 50100 Kozani, Greece; 3 Kenhub GmbH, 04318 Leipzig, Germany; 4 Department of Neurology, University Hospital of Ioannina, University of Ioannina, 45110 Ioannina, Greece

**Keywords:** spatial memory, spatial navigation, development, human, children

## Abstract

Although spatial memory has been widely studied in rodents, developmental studies involving humans are limited in number and sample size. We designed and studied the validity of two simple experimental setups for the evaluation of spatial memory and navigation development. The dataset of this study was composed of 496 schoolchildren, from 4 to 15 years old. Participants were tested blindfolded on their ability to navigate in a square area between three stool stations while performing an item-collecting task, having observed the experimental space and procedure (Test 1) or having, in addition, executed the task open-eyed (Test 2). The performance times were analyzed to identify age-specific differences. Parametric methods, including the one-way ANOVA and independent samples *t*-test, were employed. Statistically significant differences were observed in the mean performance time among age groups, as well as within the same age groups when comparing Test 1 and Test 2. Our results revealed a performance improvement with aging for both functions and showed that spatial memory and spatial navigation develop throughout childhood and puberty and interact during development. When children integrate visual stimuli with other sensory inputs, they can form stronger spatial memories, thereby enhancing their navigation skills. The proposed experimental setup is considered feasible and can be used for behavioral studies of navigation-related memory in children and beyond with appropriate adaptations, allowing for large-scale assessment.

## 1. Introduction

Space is all around us; we live in it, navigate through it, and retain memories within it. Thus, it is critical in shaping our daily experiences and interactions. The neurophysiological basis for our perception of space was largely unknown until the discovery of space cells in the hippocampus of experimental animals [1], activated by the animal’s position within the environment. Earlier studies on the famous Henry Molaison (HM) patient had already highlighted the critical role of the hippocampus in declarative memory [2]. The hippocampus is a paired structure of the central nervous system (CNS) located in each temporal lobe on the medial wall of the lateral ventricle. The hippocampal and parahippocampal areas are central structures of the limbic system [3].

### 1.1. Spatial Memory

The discovery of place cells in the hippocampus, followed by the finding of grid cells, head direction cells, and border cells, significantly advanced our understanding of spatial memory [4,5,6]. Spatial memory is organized around two major concepts, egocentric and allocentric spatial representations, both of which are essential for forming cognitive maps [7]. Egocentric representation encodes the object’s position relative to one’s body (object-to-self relation). In contrast, allocentric representation encodes the object’s position relative to other objects (landmarks). Studies have shown that the integration of place cells and grid cells, which provide metric spatial encoding, along with other specialized cells, contribute to the construction of cognitive maps [8,9,10].

The hippocampus interacts with multiple sensory systems for the perception of spatial memory and the creation of cognitive maps. Visual stimuli are the most common information source in both short-term and long-term spatial memories, with the parietal and occipital cortex playing a major role along with the hippocampal formation [11,12,13,14,15,16]. Other studies highlight the role of sound stimuli [17], proprioception [18,19], and the vestibular system [20,21] for spatial memory encoding.

### 1.2. Spatial Memory as a Cognitive Skill

Spatial memory plays a crucial role in many cognitive skills. It is involved in short-term memory, contributes to the formation of episodic memory, and serves as the foundational memory framework for spatial navigation. In Baddeley’s theory of working memory, spatial working memory is encapsulated within the visuospatial memory subsystem, and thus is referred to as visuospatial working memory (VSWM). VSWM is a specialized component of working memory responsible for the temporary storage and manipulation of visual and spatial information, allowing individuals to recall and engage with visual features (such as shapes and colors) and spatial locations (such as distances and orientations) over brief periods [22]. This capacity relies on the coordinated activity of the dorsolateral prefrontal cortex (DLPFC), the posterior parietal cortex (PPC), the occipital cortex, and the hippocampus [10,23,24,25,26].

Spatial memory is also considered a subcomponent of episodic memory. The latter constitutes a subset of declarative memory, involving the conscious recall of facts and events, along with spatial and temporal context. Therefore, it encodes information about what happened, when it happened, and where it happened. The formation and retrieval of episodic memories rely heavily on the neural circuits within the hippocampal formation [2,15,27].

Spatial navigation also appears to be highly dependent on the hippocampus [27,28], although multiple brain regions, including the entorhinal cortex [10], parietal cortex [29], prefrontal cortex [30], basal ganglia, and cerebellum, also play essential roles in this process. Spatial memory is integral to spatial navigation, with different memory systems contributing to distinct navigation strategies. Egocentric navigation relies more on short-term and working spatial memory [31,32], while allocentric navigation depends fundamentally on the hippocampus and long-term spatial memory [10,33]. The ability to use landmarks and geometrical information about space, particularly when influenced by the placement of an object, is a crucial cognitive skill for spatial navigation. This is especially significant for the spatial orientation performance of individuals with cognitive impairments like dementia [34]. The significance of spatial navigation supported by spatial memory as a cognitive skill is also reflected in its behavioral and decision-making effects on daily activities such as driving [35].

### 1.3. The Development of Spatial Memory

The perception of space changes throughout life. Typically, egocentric representation, although rudimentary, is the first type of spatial memory to develop, developing as early as in the first months of life. During the first six months of life, infants rely exclusively on egocentric spatial representation to perceive space, supporting their early, undeveloped navigation ability [36,37]. By the end of the first year, as spatial memory develops, infants begin to use landmarks and environmental cues to recognize familiar locations and navigate within small-scale environments [38,39]. Unlike egocentric representation, allocentric representation starts developing later, though the exact time point remains a topic of debate. Some studies indicate that spatial representation begins to develop at the age of two but remains rudimentary until ages four-to-five [40,41,42]. This finding aligns with other research suggesting that allocentric abilities and cognitive mapping emerge as spatial navigation strategies between the ages of two and nine [41,43,44]. However, other studies have demonstrated that allocentric representation and small-scale navigation are absent in children aged four-to-eight [45], and other research indicates that children under six cannot use viewpoint-independent allocentric strategies [46]. It is not until around the age of twelve that spatial memory, navigation, and the construction of cognitive maps begin to function similarly to adults, although this capacity continues to evolve throughout adolescence [47]. The active exploration of the environment improves spatial navigation. Children with neurodevelopmental and motor disorders often exhibit a reduced capacity for spatial memory due to limitations in active self-initiated exploration among other factors. Therefore, providing appropriate tools to assess spatial navigation early in childhood is crucial to improve intervention strategies and developmental outcomes [48].

### 1.4. Aim of This Study

Various experimental setups have been employed in the investigation of spatial memory, and despite the occasional incongruities in the obtained data there exists a consensus that the capacity to form spatial memories undergoes age-related changes [47]. Herein, we created a simple experimental setup to study the development of spatial memory and spatial navigation in children aged 4-to-15. Our objective is to devise a simple, children-friendly, and reproducible experimental protocol. The present study represents the inaugural phase in the construction of our experimental paradigm, wherein our focus is directed toward evaluating its viability of discerning developmental differentials. Therefore, we test the feasibility of the experimental setup and publish the initial conclusions from our measurements.

## 2. Materials and Methods

A total of 496 schoolchildren, 4-to-15 years old, enrolled in kindergartens, primary schools, and high schools in two cities of Greece (Ioannina and Athens) were recruited after open invitation to the public and private schools of these regions. Informed consent forms describing the experimental process and the aim of this study as well as ensuring the anonymity and confidentiality of the collected data were voluntarily filled out by all legal guardians before their child participated in the spatial memory assessment test. Human subject research was approved by the Research Ethics Committee of the University of Ioannina (No 54790/26-10-2022).

Our recruitment aimed to obtain a sample that closely reflects the demographic characteristics of the Greek population. Nonetheless, we acknowledge the potential for bias introduced by variations in regional and socioeconomic factors, which will be discussed in Section 5.

### 2.1. General Experimental Procedure

**Figure 1:**
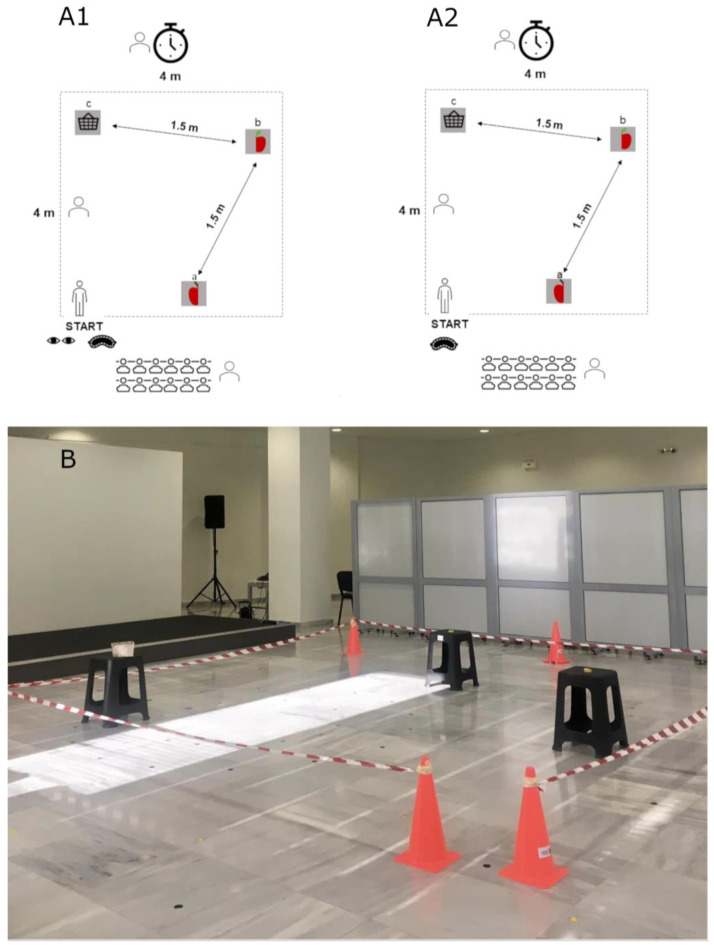
(**A**) Schematic representation of the experimental setup and procedure for the assessment of spatial memory and spatial navigation in testing room 1 (**A1**) and testing room 2 (**A2**). (**B**) Photographic depiction of the experimental setup.

The experiments took place at Serafeio Athletic and Community Complex in Athens and the “Karolos Papoulias” Conference Centre, at the University of Ioannina, as part of two-day neuroscience dissemination events with educational purposes for children, including interactive neuron microscopy and brain anatomy workshops as well as film screenings about the brain (https://www.exploringthebrain.gr/ accessed on 1 September 2024). Two testing areas (Figure 1) of 4 × 4 m^2^ were set up. The square areas were bordered with ribbons, leaving an opening as an entrance to the testing area. Three identical stools (36.5 × 36.5 × 46.0 cm) were placed in a zigzag symmetry, with a fixed distance of 1.5 m from each other. Two pieces of a self-assembled velcro wooden toy were placed on the first two stool stations (one piece on each), while the third stool station had a basket. Students arrived in groups of 10–20 (depending on the size of each school class) and were assigned to either the first test room or the second test room, but no student performed both tests. Before the main study was carried out, we conducted a small-scale pilot trial (recruiting students from one regional school only) to assess the feasibility of the experimental setup and troubleshoot unforeseen issues.

### 2.2. Data Collection

Record sheets were designed to manually record the participant’s school, grade/age, gender, observation duration, and performance time along with additional notes based on observations made by the data-entry operators/observers.

### 2.3. Testing Rooms

In each test room, we studied spatial navigation and spatial memory ability. In the first room, we conducted Test 1. After being provided with a comprehensive—adapted to their age—exposition of the experimental protocol (the test was likened to the blind man’s buff game) and making sure they had fully understood their task, i.e., to collect the pieces from the first two stool stations and place them into the basket/third stool station, students entered into the testing area one by one, blindfolded with a night mask. An observer recorded the performance time for each student and the observation of the experimental area duration. The latter recording was obtained by measuring the time from the arrival of the group of students to the time the night mask was placed on each student. The researcher who blindfolded the students made sure that they were not able to see through the mask by asking them simple questions, e.g., “Can you see my grimace?” or “How many fingers are these?”. The same researcher stayed in the testing area, following the students to ensure their security and prevent them from falling onto the borders. A third researcher supervised the group of students waiting for their turn while observing the space and experimental process. Participants undertaking the navigation test were given instructions when they were fully disoriented, panicked, or unable to complete the task. Our intervention in these cases included sound signaling (i.e., knocking on the closest stool) or verbal encouragement and direction. However, these students were excluded from the sample. In the second room, we conducted Test 2. Before conducting the task blindfolded, each student had to enter the experimental area to accomplish the task open-eyed. This way, we aspired to incorporate the experience element in the navigation test. Students were able to position themselves in the experimental environment and familiarize themselves with it before performing the close-eyed task just like in Test 1.

### 2.4. Data Analysis

**Figure 2:**
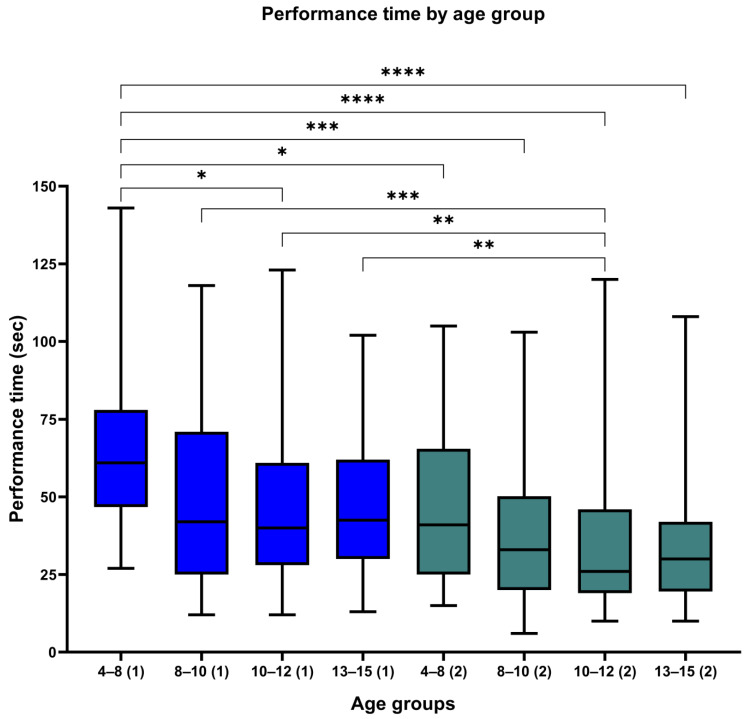
Boxplot of performance time by age group for Test 1 (indicated by (1)) and Test 2 (indicated by (2)). The level of statistical significance is indicated by the use of asterisks. A single asterisk (*) denotes a *p*-value of less than 0.05, two asterisks (**) indicate a *p*-value of less than 0.01, three asterisks (***) denote a *p*-value of less than 0.001, (****) signify a *p*-value of less than 0.0001.

Collected data were registered in Excel (Microsoft 365) and were analyzed with SPSS (Statistical Package for the Social Sciences version 29.0.1.0). We also used GraphPad Prism version 10.2.2 for the creation of the boxplot in Figure 2. Our dependent variable was the performance time (s) selected for its utility in evaluating the participants’ performance and comprehensively reflecting their memory capacity. This selection was informed by the recognition that movement speed constitutes a variable that could both influence and be influenced by the developmental trajectory of spatial memory and navigation. This reciprocal influence would introduce bidirectional relationships, complicating the interpretation of the results. For the correlation coefficients we used 0.01 as the significance level. The one-way ANOVA test and independent samples *t*-test were performed to investigate significant differences between the mean performance time of different age groups, with 0.05 as the significance level.

## 3. Results

### 3.1. Test 1

The valid size of the Test 1 sample amounted to 247 students. Table 1 summarizes the descriptive statistics of the sample. Out of the 247 participants, 133 (53.8%) were males and 114 (46.2%) were females, indicating a relatively balanced distribution between the sexes. Students’ performance time ranged from 12 to 143 s, with a mean of 49.48 s for the entire visuospatial memory cohort. When disaggregated by gender, the mean performance times were 46.08 s (95% CI: 41.86–50.29) and 53.45 s (95% CI: 48.41–58.48) for the male and female participants, respectively. Age groups were inferred from the school classes of the students, resulting in the following classifications: 4–8 years (*n* = 26, 10.5%), 8–10 years (*n* = 58, 23.5%), 10–12 years (*n* = 107, 43.5%), and 12–15 years (*n* = 56, 22.7%). These had respective mean performance times of 64.88 s (95% CI 53.27–76.50), 49.59 s (95% CI 42.19–56.18), 46.31 s (95% CI: 41.70–50.92), and 48.27 s (95% CI: 41.71–54.73). Figure 2 displays a boxplot illustrating the performance times for each age group in both Test 1 and Test 2.

Next, we examined the hypothesis that performance time improves (decreases) as students age. We performed a one-way ANOVA test (Table 2). We also performed an independent samples *t*-test to investigate whether there were significant differences in performance times between the age groups for each test. The one-way ANOVA results indicated a statistically significant difference (F(3.243) = 3.733, *p* = 0.012). The Tukey HSD test showed a statistically significant difference between the 4–8-year-old (M = 64.88, SD = 28.7) and the 10–12-year-old (M = 46.31, SD = 24.0) groups (*p* = 0.006) as well as with the 12–15-year-old (M = 48.27, SD = 24.1) group (*p* = 0.033). These findings support the hypothesis that performance time improves with age.

### 3.2. Test 2

The valid sample size for Test 2 amounted to 249 students, 136 (54.6%) of whom were males and 113 (45.4%) of whom were females, indicating a balanced distribution between the sexes. The performance time ranged from 6 s to 120 s with a mean of 36.73 s. The mean performance times were 36.52 s (95% CI: 32.43–40.62) and 36.97 s (95% CI: 32.98–40.97) for the male and female participants, respectively. Age groups were determined based on the school grades of the students, resulting in the following classifications: 4–8 years (*n* = 45, 18.1%), 8–10 years (*n* = 46, 18.5%), 10–12 years (*n* = 117, 47.0%), and 12–15 years (*n* = 41, 16.5%). The average performance time tended to decrease as age increased: 45.96 (95% CI: 38.90–53.01) for the 4–8-year-old group, 37.17 s (95% CI: 30.63–43.72) for the 8–10-year-old group, 33.43 s (95% CI: 29.43–37.42) for the 10–12-year-old group, and 35.51 s (95% CI: 27.83–43.19) for the 12–15-year-old group (Table 1).

The one-way ANOVA test, shown in Table 2, revealed again a significant effect of age on the performance time (F(3245) = 3.381, *p* =0.019), while the post hoc pairwise comparisons using the Tukey HSD test showed a lower performance in the 4–8-year-old group (M = 45.96, SD = 23.476) compared to the 10–12-year-old group (M = 33.43, SD = 21.818) (*p* = 0.010). We also compared the performance time among the same age groups for each test and the analysis revealed statistically significant differences in all comparisons, as detailed in Table 3.

### 3.3. Sex Differences

**Figure 3:**
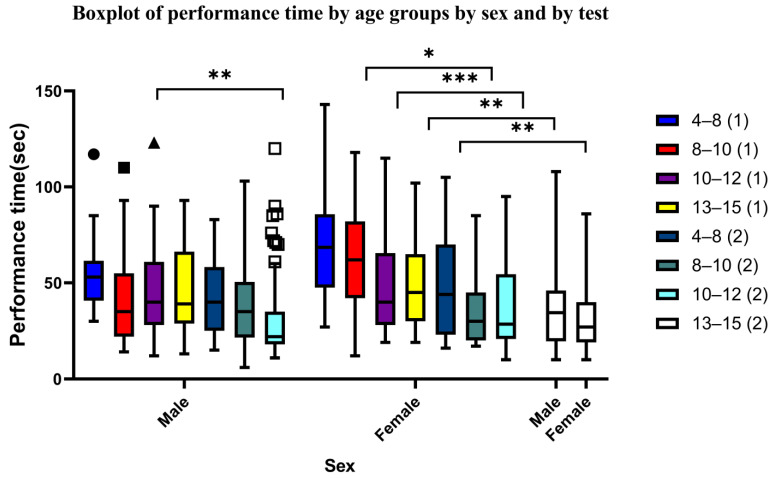
Boxplot illustrating performance across age groups for Test 1 (indicated by (1)) and Test 2 (indicated by (2)). The level of statistical significance is indicated by the use of asterisks. A single asterisk (*) denotes a *p*-value of less than 0.05, two asterisks (**) indicate a *p*-value of less than 0.01, three asterisks (***) denote a *p*-value of less than 0.001. The geometric shapes above the box plots indicate the outlier values, categorized by test and age group, respectively.

Table 4 shows the descriptive statistics for males and females across age groups for Test 1 and Test 2. We used parametric statistical methods to compare the mean performance time between males and females in each age group within each test without statistically significant differences. Therefore, focusing on male-versus-female performance in each age group yielded no substantial difference in the median performance. However, when we compared the performance of participants of the same sex and age group between Test 1 and Test 2, we found statistically significant differences for females in all age groups between Test 1 and Test 2. Females could complete Test 2 more easily than Test 1. For males, we only observed statistically significant differences between Test 1 and Test 2 in the 10–12-year-old age group (Figure 3). Additionally, females showed a rather greater inter-individual variation in performance time, as inferred by the wider ranges and interquartile ranges (IQRs) compared to males (Figure 3).

## 4. Discussion

The formation of spatial memories is contingent on all the primary cognitive functions related to space perception, including spatial navigation. Spatial memory not only supports spatial navigation [31], but is also reinforced by it, as navigating helps in forming new spatial memories and cognitive maps [49]. Currently, there is extensive literature on spatial memory in experimental animals, but data on human behavior, especially during development, remain sparse and incomplete. The primary goal of the present study is to propose a simple and children-friendly procedure assessing the formation of spatial memories and spatial navigation and to validate this procedure in a large dataset. Furthermore, we aim to enhance our understanding of the development of spatial memory and navigation in childhood and puberty.

We designed our study to contain two distinct experimental procedures. The first one (Test 1) was a spatial navigation test based on visual stimuli, which seem to serve as the raw material for both spatial egocentric and allocentric representations of the room [50,51]. When blindfolded, the succession of tasks relies heavily on navigation strategies, which are fundamentally dependent on spatial memory [31]. In the second procedure (Test 2), children primarily used spatial navigation strategies with their eyes open, but when blindfolded they also relied on their spatial memory to succeed. Thus, in Test 1 we focused on the creation of spatial memories using visual cues and landmarks, while in Test 2 we examined the formation of spatial memories through navigation in the room.

Based on the performance time results among different age groups in each task, we conclude that the ability to use spatial memory for navigation develops throughout the ages tested (from 4 to 15 years old). There are statistically significant differences between distinct age groups (4–8, 8–10, and 13–15 for Test 1 and 4–8 and 10–12 for Test 2), indicating that spatial development occurs gradually. Children can use visual stimuli to create spatial memories and apply them for navigation, an ability that evolves over time. This aligns with the existing literature indicating significant age marks of developmental changes in the hippocampus volume [49,52] and functional hippocampal networks [53] as well as their connections with other brain regions, including the prefrontal cortex [54], thus playing a crucial role in spatial memory and navigation. The results of Test 1 reflect that spatial memory plays a critical role in supporting spatial navigation when an individual enters a new environment for the first time. This ability appears to develop with age, as older children completed the test more efficiently. Test 2 results are in line with the fact that spatial navigation, a process enriched by multiple sensory inputs (including visual, proprioceptive, and motor stimuli), contributes to the formation of spatial memories. These memories, in turn, enhance the understanding of space and the ability to navigate effectively within it.

The interplay between spatial memory and navigation has significant implications in clinical contexts. Research indicates that conditions such as neurodevelopmental disorders (e.g., cerebral palsy) and motor disorders are often associated with an inability to actively explore the environment, leading to deterioration of spatial memory and navigation abilities [50]. In such cases, motor challenges, such as impairments in gait speed, stability, gaze anticipation, and head–trunk coordination, further exacerbate these difficulties. For example, children with cerebral palsy, as well as those with visuospatial memory deficits [51,55,56,57,58], demonstrate altered spatial memory and navigation abilities compared to neurotypical children due to their limited capacity for active exploration and reduced exposure to diverse stimuli. Moreover, even among neurotypical children socioeconomic and regional factors can influence the complexity of environmental cues, which in turn affects the development of their spatial abilities [59,60]. However, it is difficult to identify which specific aspects of spatial memory (e.g., egocentric, allocentric, cognitive maps) play crucial roles in each task or to determine the spatial navigation strategies (e.g., egocentric/allocentric navigation, path integration, landmark-based navigation) involved. We believe that future studies could provide deeper insights into all facets of spatial memory and navigation by manipulating parameters within our experimental procedure. Our setup is designed to investigate the development of the entire spatial memory and navigation system rather than isolated components. Future research could also adapt our setup to focus on specific aspects of these cognitive skills.

Spatial memory abilities begin to emerge in infancy, but noticeable sex differences have often been described as appearing after the age of four-to-six years [61]. Our study was not specifically designed to measure sex differences (see Limitations). However, our statistical analysis indicates that females performed better in Test 2 compared to Test 1. This observation suggests that males and females may exhibit strengths and weaknesses in different aspects of spatial memory and navigation. Boys generally demonstrate a greater proficiency in spatial reasoning tasks, while girls excel in spatial memory tasks involving verbal labeling and object recognition [62]. Hormonal changes during puberty, particularly increases in testosterone in males and estrogen in females, are also thought to influence hippocampal function and spatial abilities [63].

The experimental method we used is advantageous in terms of cost-effectiveness and ethical approval. Contrary to many similar studies, a large sample size was acquired and thoroughly analyzed. Furthermore, the game-like experimental design along with the accompanying activities provided an enjoyable, educational, and engaging experience for children, making participation accessible to a wider audience. Nonetheless, further research is needed to refine the proposed experimental procedure. Future investigations should explore how variables other than age impact the development of spatial memory. For instance, manipulating inter-object distances, stool heights, and the spatial arrangement of targets could enhance our understanding of their effects on navigation capacity and the development of spatial memory.

Future studies should concentrate on the refinement of the experimental procedure. First, it is necessary to refine various variables, like the dimensions of the test area, based on the participants’ age and body composition. Also, the experimental procedure can be modified to reflect distinct aspects of spatial memory (e.g., egocentric, allocentric representation) and spatial navigation (e.g., navigation strategies like path integration). Such attempts can pave the way to behaviorally studying specific brain regions and circuits as well as their role in navigation-associated cognitive abilities and provide insight in how different cognitive aspects of navigation are affected by the spatial memory across different developmental stages. This can be achieved through either minor adjustments or more extensive modifications to our basal experimental procedure. For example, the use of electroencephalography (EEG) during the tests could offer valuable insight. The continuous movement of children, though, does not facilitate the use of EEG. This limitation could be addressed by the deployment of a virtual reality (VR) environment, which allows for greater control and feasibility. Additionally, future studies could focus on young age groups, such as four-to-eight years, where our study had fewer participants, and investigate potential sex differences in spatial navigation and memory.

## 5. Limitations

In this article, we examined the development and interaction of spatial memory and spatial navigation. This investigation provides a general overview of the development of these two abilities, but it is not without limitations. First, there is potential bias in the participant recruitment process due to regional and socioeconomic factors. Most participants were recruited from larger urban areas, although we made efforts to obtain a representative sample of the Greek population demography. Second, this study included fewer participants in the four-to-eight age range compared to other age groups. Recruiting younger participants can be challenging due to parental consent requirements and a child’s capacity to complete tasks. Consequently, a detailed analysis of this age range was not statistically meaningful. For comparisons involving the other two-year age groups (i.e., the 8–10-, 10–12-, and 13–15-year-old groups), the 4–8-year-old group encompassed a broader age span, which may have introduced variability. The aim of the present study was capturing general trends rather than precise developmental differences. Therefore, we accepted a more heterogenous four-to-eight-year-old group to explore behavioral trends in young children. This study might serve as a stepping stone for more refined future research targeting narrower ranges. In addition, this study was not designed to investigate sex differences. Some age groups lacked a balanced sample size of male and female participants. Moreover, there are numerous factors influencing sex differences, including cultural and educational status, as well as biological factors such as hormone levels (e.g., testosterone, estrogens), the measurement of which was beyond the scope of this study.

It remains unclear whether body type significantly influences these assessments. On one hand, older children possess a body type that allows for faster movement and easier task completion. On the other hand, being blindfolded necessitates a better understanding of spatial relationships to maintain velocity and balance [20,21]. Thus, it is not solely physical changes throughout child development that may confer advantages in navigation or spatial memory, but rather a combination of various characteristics [52]. By comparing the performance times across tasks within the same age groups—where body types are assumed to be similar—we conclude that employing navigation strategies alongside other stimuli (vision, proprioception) facilitates movement when blindfolded, indicating that children possess better spatial memories of the room. Children can integrate various environmental stimuli to create more accurate spatial memories, enhancing their navigation skills, which develop as they grow older.

## 6. Conclusions

We conclude that the experimental setup we proposed herein is feasible, cost-effective, and reproducible. It supports several long-established theories regarding the development of spatial memory and other cognitive skills within the existing scholarly discourse. Our findings also suggest that the ability to create spatial memories and utilize them for navigation develops with age and they are more adept at this task when they engage with multiple environmental stimuli within a room.

## Figures and Tables

**Table 1:** Test 1 (left panel) and Test 2 (right panel) performance time (s) descriptive statistics by age group.

	Test 1				Test 2			
Variable	*n* (%)	M	SD	CI (95%)	*n* (%)	M	SD	CI (95%)
**Total**	247 (100)	49.48	26.0		249 (100)	36.73	22.9	
**4–8**	26 (10.5)	64.88	28.7	53.27–76.50	45 (18.1)	45.96	23.4	38.90–53.01
**8–10**	58 (23.5)	49.59	28.1	42.19–56.98	46 (18.5)	36.87	22.1	30.20–43.53
**10–12**	107 (43.3)	46.31	24.0	41.70–50.92	117 (47.0)	33.43	21.8	29.43–37.42
**13–15**	56 (22.7)	48.27	24.1	41.81–54.73	41 (16.5)	35.51	24.3	27.83–43.19

**Table 2:** One-way ANOVA results for the mean performance time of the different age groups in Test 1 (left panel) and Test 2 (right panel).

One-Way ANOVA Test 1							One-Way ANOVA Test 2						
Performance Time (s)							Performance Time						
	Sum of Squares	df	Mean Square	F	Sig.			Sum of Squares	df	Mean Square	F	Sig.	
Between Groups	7329.10	3.00	2443.03	3.73	0.01		Between Groups	5176.03	3.00	1725.35	3.38	0.02	
Within Groups	159,036.53	243.00	654.47				Within Groups	125,021.40	245.00	510.29			
Total	166,365.63	246.00					Total	130,197.43	248.00				
**Multiple Comparisons**							**Multiple Comparisons**						
Dependent Variable:							Dependent Variable:						
**Tukey HSD**							**Tukey HSD**						
(I) Age Groups		Mean Difference (I-J)	Std. Error	Sig.	95% Confidence Interval		(I) Age Groups		Mean Difference (I-J)	Std. Error	Sig.	95% Confidence Interval	
					Lower Bound	Upper Bound						Lower Bound	Upper Bound
4–8	8–10	15.30	6.04	0.06	−0.32	30.92	4–8	8–10	8.78	4.74	0.25	−3.47	21.03
	10–12	18,576 *	5.59	0.01	4.11	33.05		45,636.00	12,528 *	3.96	0.01	2.28	22.78
	12–15	16,617 *	6.07	0.03	0.91	32.32		12–15	10.44	4.88	0.14	−2.17	23.06
8–10	4–8	−15.30	6.04	0.06	−30.92	0.32	8–10	4–8	−8.78	4.74	0.25	−21.03	3.47
	10–12	3.28	4.17	0.86	−7.51	14.07		10–12	3.75	3.93	0.78	−6.42	13.92
	12–15	1.32	4.79	0.99	−11.08	13.72		12–15	1.66	4.85	0.99	−10.89	14.21
10–12	4–8	−18,576 *	5.59	0.01	−33.05	−4.11	10–12	4–8	−12,528 *	3.96	0.01	−22.78	−2.28
	8–10	−3.28	4.17	0.86	−14.07	7.51		8–10	−3.75	3.93	0.78	−13.92	6.42
	12–15	−1.96	4.22	0.97	−12.87	8.96		12–15	−2.08	4.10	0.96	−12.69	8.52
12–15	4–8	−16,617 *	6.07	0.03	−32.32	−0.91	12–15	4–8	−10.44	4.88	0.14	−23.06	2.17
	8–10	−1.32	4.79	0.99	−13.72	11.08		8–10	−1.66	4.85	0.99	−14.21	10.89
	10–12	1.96	4.22	0.97	−8.96	12.87		10–12	2.08	4.10	0.96	−8.52	12.69

* The mean difference is significant at the 0.05 level.

**Table 3:** Independent samples *t*-test for performance time between age groups for each test.

Independent Samples Test Between Performance Time in the Same Age Groups of Each Test										
**Age Groups 4–8**	Levene’s Test *		*t*-test for Equality of Means							
	F	Sig.	*t*	df	Significance		Mean Difference	Std. Error Difference	95% Confidence Interval of the Difference	
					One-Sided *p*	Two-Sided *p*			Lower	Upper
Equal Variances Assumed	0.383	0.538	3.011	69	0.002	0.004	18.92906	6.28626	6.38832	31.4698
Equal Variances not Assumed			2.851	44.226	0.003	0.007	18.92906	6.63829	5.5524	32.30572
**Age Groups 8–10**	Levene’s Test *		*t*-test for Equality of Means							
	F	Sig.	*t*	df	Significance		Mean Difference	Std. Error Difference	95% Confidence Interval of the Difference	
					One-Sided *p*	Two-Sided *p*			Lower	Upper
Equal Variances Assumed	7.445	0.007	2.455	102	0.008	0.016	12.41229	5.05652	2.38271	22.44188
Equal Variances not Assumed			2.524	101.991	0.007	0.013	12.41229	4.91778	2.65789	22.16669
**Age Groups 10–12**	Levene’s Test *		*t*-test for Equality of Means							
	F	Sig.	*t*	df	Significance		Mean Difference	Std. Error Difference	95% Confidence Interval of the Difference	
					One-Sided *p*	Two-Sided *p*			Lower	Upper
Equal Variances Assumed	1.222	0.27	4.204	222	<0.001	<0.001	12.88106	3.06415	6.84252	18.9196
Equal Variances not Assumed			4.186	214.572	<0.001	<0.001	12.88106	3.07748	6.8151	18.94702
**Age Groups 13–15**	Levene’s Test *		*t*-test for Equality of Means							
	F	Sig.	*t*	df	Significance		Mean Difference	Std. Error Difference	95% Confidence Interval of the Difference	
					One-Sided *p*	Two-Sided *p*			Lower	Upper
Equal Variances Assumed	0.761	0.385	2.562	95	0.006	0.012	12.75566	4.97828	2.87252	22.6388
Equal Variances not Assumed			2.559	85.926	0.006	0.012	12.75566	4.98496	2.84577	22.66556

* Levene’s Test for Equality of Variances.

**Table 4:** Test 1 (up) and Test 2 (down) Performance Time (sec) Descriptive Statistics.

	N	Mean	Std. Deviation	Std. Error	95% Confidence Interval for Mean		Minimum	Maximum
					Lower Bound	Upper Bound		
Test 1								
Male 4–8 (1)	13	58.4615	23.10705	6.40874	44.4981	72.425	30	117
Female 4–8 (1)	13	71.3077	33.16973	9.19963	51.2634	91.352	27	143
Male 8–10 (1)	35	42.8286	26.56234	4.48986	33.7041	51.9531	14	110
Female 8–10 (1)	23	59.8696	27.82299	5.80149	47.838	71.9011	12	118
Male 10–12 (1)	59	45.1017	23.33393	3.03782	39.0208	51.1825	12	123
Female 10–12 (1)	48	47.7917	25.05395	3.61623	40.5168	55.0666	19	115
Male 13–15 (1)	26	46.4615	24.77455	4.85869	36.4549	56.4682	13	93
Female 13–15 (1)	30	49.8333	23.87335	4.35866	40.9189	58.7478	19	102
Test 2								
Male 4–8 (2)	22	44	20.00714	4.26554	35.1293	52.8707	15	83
Female 4–8 (2)	23	47.8261	26.69636	5.56658	36.2817	59.3705	16	105
Male 8–10 (2)	25	39.28	25.35008	5.07002	28.816	49.744	6	103
Female 8–10 (2)	21	34.6667	17.59072	3.83861	26.6595	42.6739	17	85
Male 10–12 (2)	71	32.2113	22.97944	2.72716	26.7721	37.6504	11	120
Female 10–12 (2)	46	35.3043	19.99319	2.94783	29.3671	41.2416	10	95
Male 13–15 (2)	18	40.5556	29.5699	6.96969	25.8508	55.2603	10	108
Female 13–15 (2)	23	31.5652	19.09028	3.9806	23.31	39.8205	10	86
Total	496	43.0766	25.29515	1.13579	40.8451	45.3082	6	143

## Data Availability

The data and materials for all experiments are available upon reasonable request.
